# P2RX7 Deletion in T Cells Promotes Autoimmune Arthritis by Unleashing the Tfh Cell Response

**DOI:** 10.3389/fimmu.2019.00411

**Published:** 2019-03-19

**Authors:** Krysta M. Felix, Fei Teng, Nicholas A. Bates, Heqing Ma, Ivan A. Jaimez, Kiah C. Sleiman, Nhan L. Tran, Hsin-Jung Joyce Wu

**Affiliations:** ^1^Department of Immunobiology, University of Arizona, Tucson, AZ, United States; ^2^Arizona Arthritis Center, College of Medicine, University of Arizona, Tucson, AZ, United States

**Keywords:** P2RX7, autoimmune, microbiota, apoptosis, TIGIT

## Abstract

Rheumatoid arthritis (RA) is an autoimmune disease that affects ~1% of the world's population. B cells and autoantibodies play an important role in the pathogenesis of RA. The P2RX7 receptor is an ATP-gated cation channel and its activation results in the release of pro-inflammatory molecules. Thus, antagonists of P2RX7 have been considered to have potential as novel anti-inflammatory therapies. Although originally identified for its role in innate immunity, P2RX7 has recently been found to negatively control Peyer's patches (PP) T follicular helper cells (Tfh), which specialize in helping B cells, under homeostatic conditions. We have previously demonstrated that PP Tfh cells are required for the augmentation of autoimmune arthritis mediated by gut commensal segmented filamentous bacteria (SFB). Thus, we hypothesized that P2RX7 is required to control autoimmune disease by keeping the Tfh cell response in check. To test our hypothesis, we analyzed the impact of P2RX7 deficiency *in vivo* using both the original K/BxN autoimmune arthritis model and T cell transfers in the K/BxN system. We also examined the impact of P2RX7 ablation on autoimmune development in the presence of the gut microbiota SFB. Our data illustrate that contrary to exerting an anti-inflammatory effect, P2RX7 deficiency actually enhances autoimmune arthritis. Interestingly, SFB colonization can negate the difference in disease severity between WT and P2RX7-deficient mice. We further demonstrated that P2RX7 ablation in the absence of SFB caused reduced apoptotic Tfh cells and enhanced the Tfh response, leading to an increase in autoantibody production. It has been shown that activation of TIGIT, a well-known T cell exhaustion marker, up-regulates anti-apoptotic molecules and promotes T cell survival. We demonstrated that the reduced apoptotic phenotype of *P2rx7*^−/−^ Tfh cells is associated with their increased expression of TIGIT. This suggested that while P2RX7 was regulating the Tfh population by promoting cell death, TIGIT may have been opposing P2RX7 by inhibiting cell death. Together, these results demonstrated that systemic administration of general P2RX7 antagonists may have detrimental effects in autoimmune therapies, especially in Tfh cell-dependent autoimmune diseases, and cell-specific targeting of P2RX7 should be considered in order to achieve efficacy for P2RX7-related therapy.

## Introduction

Rheumatoid arthritis (RA) is an autoimmune disease that causes chronic inflammation of the joints and affects ~1% of the world's population. Genetics play an important role in RA and the disease concordance rate between monozygotic twins with RA is ~15%, (1, 2). This suggests that other factors are also critically involved in RA pathogenesis, including smoking, gender, and a more recently discovered factor, the composition of the gut microbiota (3–6). Autoantibodies (auto-Abs) play critical roles in the pathogenesis of RA as they contribute to immune complex formation and complement activation, leading to tissue damage in the joints (7–12). T follicular helper (Tfh) cells are a subset of CD4^+^ T cells that co-expresses high levels of the inhibitory co-receptor PD-1 and the chemokine receptor CXCR5 (13–15). The function of Tfh cells is to help germinal center B cells produce high-titer, high-affinity, isotype-switched antibodies (Abs) and differentiate into long-lived plasma cells. Therefore, an excessive Tfh cell response can lead to over-productive auto-Ab responses and autoimmune conditions including RA (16).

High concentrations of extracellular ATP, such as those released by dying cells, can act as a danger signal by binding to the P2RX7 purinergic receptor, an ATP-gated cation channel (17). This activates the NLRP3 inflammasome pathway, which ultimately leads to the maturation and release of the pro-inflammatory cytokines IL-1β and IL-18 (18). Therefore, P2RX7 has been targeted as a means of developing anti-inflammatory therapies in many autoimmune diseases (19). However, studies examining the function of P2RX7 in autoimmunity using P2RX7 deficient mouse models suggest a complicated role for P2RX7, as P2RX7 deficiency can either suppress or exaggerate disease phenotypes (20–22). In the context of rheumatoid arthritis, there have been several clinical trials using P2RX7 antagonists as a means of reducing inflammation (19, 23, 24). However, while early results appeared to support the beneficial effects of such treatment, P2RX7 antagonists failed to improve arthritic symptoms in two recent RA clinical trials (23, 24). Understanding the mechanisms by which P2RX7 impacts autoimmunity will provide the knowledge required to properly target P2RX7 for therapeutic purposes. This is an urgent task, since despite some initial setbacks, enthusiasm for P2RX7 antagonist therapy remains strong, as evidenced by the numerous ongoing clinical trials with new P2RX7 agonist and antagonist compounds (17, 19, 25).

Interestingly, although originally identified for its crucial role in the innate immune response (19), P2RX7 has recently been found to also play key roles in regulating T cell populations. A pioneer report has shown that P2RX7 activation negatively controls Tfh cell numbers in Peyer's Patches (PPs), a type of gut-associated lymphoid tissue, to promote host-microbiota mutualism under homeostatic conditions (26). Additionally, P2RX7 drives Th1 cell differentiation and controls the follicular helper T cell population to protect against *Plasmodium chabaudi* malaria (27). However, the role of P2RX7 in the Tfh cell response under autoimmune conditions is not known. Importantly, with regard to inflammatory arthritis, a study found that 2 of 9 patients with systemic juvenile idiopathic arthritis had loss-of-function variants in *P2rx7* (28). Therefore, we hypothesized that P2RX7 deficiency enhances autoimmune disease by increasing the Tfh cell response. We have previously demonstrated that the gut microbiota constituent segmented filamentous bacteria (SFB) promote autoimmune arthritis via inducing PP Tfh cells (29). Therefore, we also examined the impact of P2RX7 ablation on autoimmune development in the presence of gut microbiota SFB.

Here, we use the K/BxN [KRN T cell receptor (TCR) transgenic mice on the C57/BL6 (B6) background x NOD] model to test our hypothesis. The K/BxN model is a murine autoimmune arthritis model in which KRN T cells recognize glucose-6-phosphate isomerase (GPI), the self-antigen presented by MHC class II I-A^g7^ from NOD mice (30). These activated T cells can in turn activate B cells to produce anti-GPI auto-Abs. K/BxN mice share many clinical and histologic features with human RA patients (31). As in many human autoimmune diseases including RA, auto-Abs play important pathological roles in K/BxN disease development (31). An advantage of the K/BxN model is that it has an easily distinguishable initial T-B cell interaction phase and a later effector phase involving innate immune players that allows for a straightforward analysis of the immune response (32–34). Thus, the intrinsic role of T cells can be easily dissected out by using the K/BxN T cell transfer model. This is done by transferring K/BxN T cells into T cell-deficient mice that express MHC II I-A^g7^ (30, 35). This approach allows for the examination of T cell-specific P2RX7 contributions and avoids many confounding effects from genetic modification of whole animals. Here we demonstrated that P2RX7 deficiency in the whole mouse caused augmented autoimmune arthritis, but SFB colonization does not further exacerbate disease in P2RX7-deficient K/BxN mice, as it does in wild type (WT) K/BxN mice. Interestingly, the arthritis enhancement in SFB(–) mice was reproducible simply by deleting P2RX7 in T cells, which led to an enhanced Tfh cell response. Thus, unlike the anti-inflammatory effect of P2RX7 blockade in innate immunity reported previously, our results indicated that P2RX7 deletion in T cells actually enhances autoimmunity by unleashing the Tfh cell response.

## Materials and Methods

### Mice

KRN TCR transgenic mice in the C57BL/6 (B6) background (KRN), TCRα^−/−^.B6, and TCRα^−/−^.NOD mice were originally obtained from the mouse colony of Drs. Diane Mathis and Christophe Benoist at the Jackson Laboratory (Jax). K/BxN mice were generated by crossing KRN mice to NOD mice (All K/BxN experimental mice are the F1 offspring of KRN and NOD parents). *P2rx7*^−/−^.B6 and *P2rx7*^−/−^.NOD mice were purchased from Jax. *P2rx7*^−/−^.B6 were crossed to KRN mice to generate *P2rx7*^−/−^.KRN mice. *P2rx7*^−/−^.K/BxN mice were generated by crossing *P2rx7*^−/−^.KRN (in B6 background) to *P2rx7*^−/−^.NOD mice. TCRα^−/−^.BxN mice were generated by crossing TCRα^−/−^.B6 with TCRα^−/−^.NOD mice. TCRα^−/−^.BxN mice thus bear the NOD MHC class II, I-A^g7^, for self-Ag presentation. Age- and gender-matched K/BxN mice between 5 and 7 weeks old were used in experiments. The K/BxN autoimmune model is a T cell receptor transgenic model, therefore, its disease development is robust and occurs in 100% of the F1 (KRNxNOD) offspring, regardless of gender (32). Additionally, we compared arthritis development between male and female K/BxN mice and found no significant difference in disease severity (Supplementary Figure 1). For transfer experiments, mice were given transfer cells at 6 weeks old, and analysis was performed 2 weeks after transfer. Ankle thickness was measured with a caliper (J15 Blet micrometer) as described previously (36). All mice were housed at the SPF animal facility at the University of Arizona. All experiments were conducted in accordance with the guidelines of the University of Arizona Institutional Animal Care and Use Committee Protocol number 11-278.

### Antibodies and Flow Cytometry

For surface staining, fluorophore-conjugated mAbs specific for CD4 (RM4-5), CD19 (6D5), CD25 (PC61), CD11c (N418), CD11b (M1/70), Gr-1 (RB6-8C5), CD45 (30-F11), TCRβ (H57-597), PD-1 (RMP1-30), CD8α (53-6.7), CD3 (17A2), and TIGIT (1G9) were obtained from BioLegend. Abs recognizing CXCR5 (2G8) were from BD Pharmingen. Abs recognizing P2RX7 (Hano43) were from Novus Biologicals. For intranuclear staining, buffers from a Foxp3 Staining Buffer Set (eBioscience) were used to stain with Abs recognizing Bcl-6(K112-91, BD Pharmingen) and Foxp3 (FJK-16s, eBioscience). For the cell death assay, cells were stained with Live/Dead® Yellow Dye (LifeTechnologies) and then surface markers, and Annexin V binding accomplished using the Annexin V/Dead Cell Apoptosis Kit (LifeTechnologies) according to the manufacturer's instructions. Cells were run on an LSRII (BD Biosciences), and analyses were performed with FlowJo (TreeStar) software.

### ELISA

Anti-GPI Ab titers were measured as described (36). Briefly, ELISA plates were coated with recombinant mouse GPI at 5 μg/ml, and diluted mouse sera were added. Subsequently, plates were washed and alkaline-phosphatase (AP)-conjugated anti-mouse IgG Abs (Jackson ImmunoResearch) were added. After the final wash, AP substrate was added and titers were quantified as optical density values via an ELISA reader. Ab titers were expressed as arbitrary units, which were calculated from serial dilutions of sample serum and defined as the reciprocal of the highest dilution that gave an above background O.D. value set as 0.15.

### Cell Transfer and Serum Transfer

T cells were transferred as described previously (35). Briefly, splenic CD4^+^ T cells (5x10^5^) were enriched by CD4-conjugated MACS beads from K/BxN or *P2rx7*^−/−^.K/BxN mice and adoptively transferred by retro-orbital (r.o.) injection into 6 week old SFB(–) or SFB(+) *Tcra*^−/−^.BxN recipients. SFB status was manipulated by gavaging recipient mice with SFB prior to cell transfer. At the indicated time point after transfer, tissues from recipient mice were harvested for flow cytometry analysis and sera were collected for ELISA.

### Microbiota Reconstitution and Quantification

Our SPF mouse colony was derived as previously described (29). SFB(–) *Tcra*^−/−^.BxN mice were weaned at 21 days old and rested for 1 day. Then, mice were orally gavaged for 3 consecutive days starting at 22 days old. SFB(–) mice were the ungavaged littermate controls. The SFB colonization status was examined 10 days after the first gavage by SFB-specific 16S rRNA quantitative PCR (36). To determine the change in SFB induced by transfer of *P2rx7*^−/−^.K/BxN T cells, fecal samples were also collected at the time of analysis, 2 weeks after the T cell transfer, and SFB colonization was examined by quantitative PCR.

### Statistical Analysis

Asterisks indicate statistical significance. Differences were considered significant when *P* < 0.05 by Student's *t*-test (two-tailed, unpaired, with Welch's correction) or one-way analysis of variance (ANOVA) followed by Tukey's multiple comparisons. To compare ankle thickening, the area under the curve (AUC) was calculated for each mouse within an experimental set followed by a Student's *t*-test between the groups (Prism 6, Graph-Pad Software). ^*^P < 0.05, ^**^P < 0.01, ^***^P < 0.001, ^****^P < 0.0001.

## Results

### P2RX7 Deficiency Enhances Autoimmune Arthritis Development

**Figure 1:**
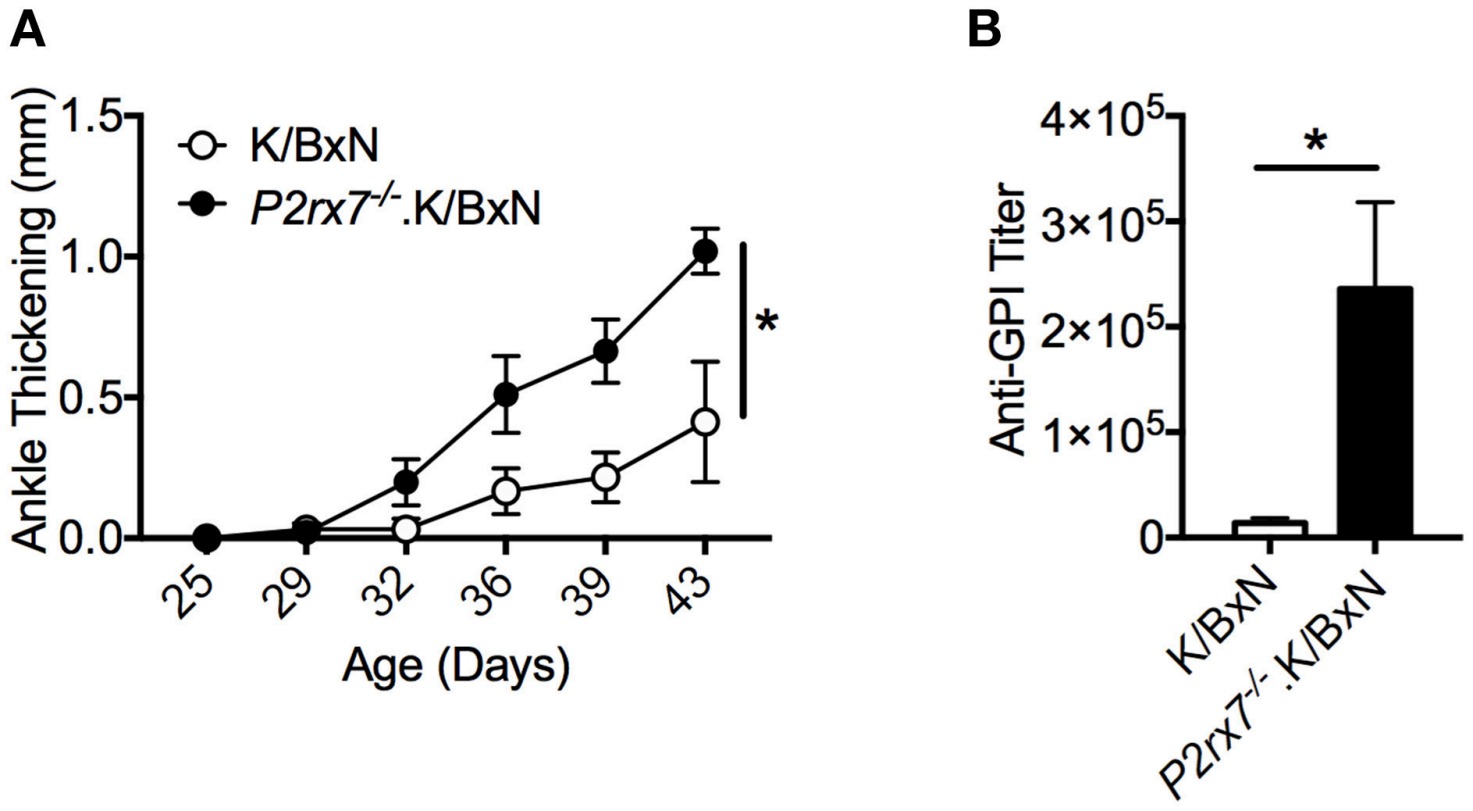
P2RX7 deficiency leads to enhanced disease development in the SFB(–) K/BxN model. **(A)** Ankle thickness was followed over time for K/BxN and *P2rx7*^−/−^.K/BxN mice between ~3.5 and 6 weeks of age. Data are shown as change in ankle thickness compared to first measurement. *N* = 9–14/group, 6 assays combined. **(B)** Anti-GPI auto-Ab titers in serum obtained from the end point of each experiment were measured by ELISA. *N* = 4–8/group, 6 assays combined. Error bars represent SEM. ^*^p < 0.05.

We first determined the role of P2RX7 in the spontaneous K/BxN autoimmune arthritis model. Genetic P2RX7 deletion (*P2rx7*^−/−^ mice) in other autoimmune models has generated results ranging from suppression, to no change, to exacerbation of the autoimmune response compared to WT controls (20–22). It is worth mentioning that some of these studies were not using fully backcrossed mice and alternative P2RX7 functions due to allelic differences among common inbred strains have been reported (37–40). Due to the complexity of P2RX7, the WT K/BxN and *P2rx7*^−/−^.K/BxN used in this study were in a fixed B6xNOD background. We found enhanced arthritis development in *P2rx7*^−/−^ compared to WT K/BxN mice at an early age, 4–6 weeks old (Figure 1A). In the K/BxN autoimmune arthritis model, disease development is driven largely by anti-GPI auto-Abs (30). Therefore, we examined the anti-GPI auto-Ab titer in *P2rx7*^−/−^ compared to WT K/BxN mice. The increased susceptibility to disease development in *P2rx7*^−/−^ mice corresponded with an increased auto-Ab response (Figure 1B). In the K/BxN model, therefore, complete P2RX7 deficiency promoted an enhanced auto-Ab response, provoking early disease onset compared to WT controls. There was no significant change in T cell development in the thymus and we also did not find any difference in peripheral immune populations including CD4^+^ T cells and CD19^+^ B cells in spleen and PPs between K/BxN and *P2rx7*^−/−^.K/BxN mice (Table 1). Furthermore, no difference was found in the innate populations including Gr-1^+^CD11b^+^ neutrophils and CD11c^+^ or CD11b^+^ antigen presenting cells in spleen between K/BxN and *P2rx7*^−/−^.K/BxN mice (Table 1).

**Table 1:** Comparison of major cell groups in K/BxN and *P2rx7*^−/−^.K/BxN mice.

**Organ**	**Cell group**	**K/BxN**	***P2rx7^**−/−**^*.K/BxN**
Thymus	CD8, CD4 DN (% of TCRβ+)	14.57 ± 1.92	13.10 ± 2.14
	CD8, CD4 DP (% of TCRβ+)	76.90 ± 2.86	78.10 ± 3.97
	CD8 SP (% of TCRβ+)	1.15 ± 0.15	1.26 ± 0.34
	CD4 SP (% of TCRβ+)	1.08 ± 0.16	0.88 ± 0.13
Spleen	CD4^+^ (% of splenocytes)	3.10 ± 0.22	3.47 ± 0.20
	CD19^+^ (% of splenocytes)	73.32 ± 1.15	71.42 ± 1.20
	CD11c^+^ (% of CD19-TCRβ-Gr-1-)	10.14 ± 1.69	10.08 ± 1.62
	CD11b^+^ (% of CD19-TCRβ-Gr-1-)	28.05 ± 2.25	30.59 ± 4.22
	Gr-1^+^CD11b^+^ neutrophils (% of CD19-TCRβ-)	16.69 ± 0.22	21.82 ± 3.47
PPs	CD4+ (% of splenocytes)	3.11 ± 0.32	3.69 ± 0.35
	CD19+ (% of splenocytes)	66.48 ± 1.420	59.37 ± 1.861

### Differing Impacts of P2RX7 Deficiency on Systemic and PP Tfh Responses

**Figure 2:**
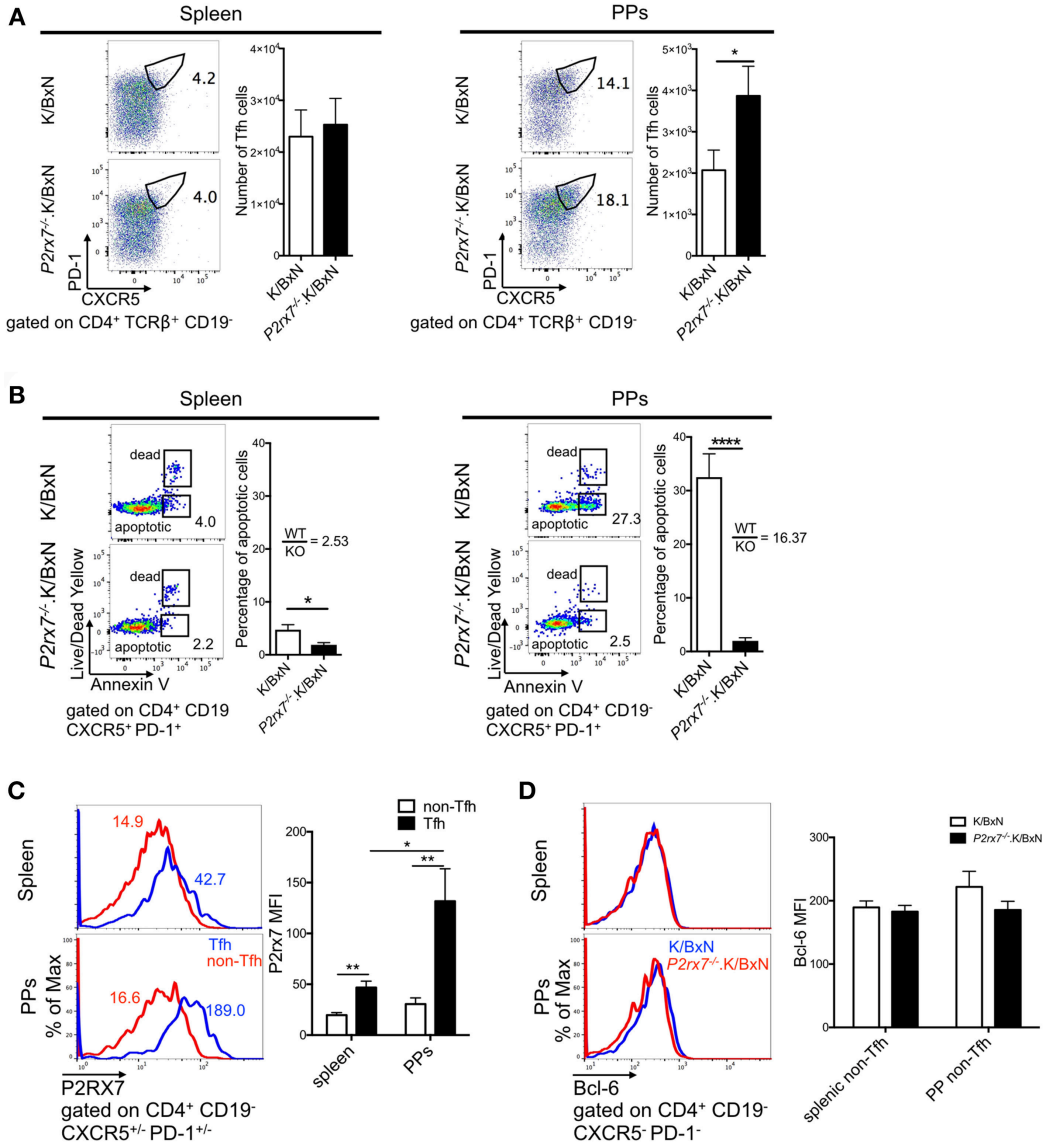
P2RX7 deletion induces the Tfh cell response in PPs but not spleen of the SFB(–) K/BxN mice. **(A)** Splenocytes and PP cells from 5 to 6 week old K/BxN and *P2rx7*^−/−^.K/BxN mice were stained with Abs against CD4, CD19, PD-1, and CXCR5. Values in the representative plots indicate the percentage of Tfh cells in total CD4^+^ T cells. Compiled graphs of the number of Tfh cells are also shown as mean + SEM. *N* = 17–18/group, 8 independent assays combined. **(B)** Tfh cells from spleen and PPs of K/BxN and *P2rx7*^−/−^.K/BxN mice were analyzed for Annexin V binding among Live/Dead Yellow^−^ cells. Representative plots and compiled graphs are shown. Ratios calculated by dividing the average of the K/BxN (WT) group by the average of the *P2rx7*^−/−^.K/BxN (KO) group. *N* = 6–7/group, 4 independent assays combined. **(C)** P2RX7 expression was detected by flow cytometry on non-Tfh and Tfh cells from spleen and PPs of K/BxN and *P2rx7*^−/−^.K/BxN mice. Representative plots and compiled graphs are shown. *N* = 8–9/group, 3 independent assays combined. **(D)** Bcl-6 expression was detected by flow cytometry on non-Tfh cells from spleen and PPs of K/BxN and *P2rx7*^−/−^.K/BxN mice. Representative plots and compiled graphs are shown. *N* = 13–17/group, 6 independent assays combined. Error bars represent means + SEM. ^*^p < 0.05, ^**^p < 0.01, ^****^p < 0.0001.

Next, we aimed to determine the cellular mechanism that caused the increased arthritis in *P2rx7*^−/−^.K/BxN mice. P2RX7 deficiency has been shown to increase PP Tfh cells in C57BL/6 (B6) mice, leading to an increase in IgA production in the PPs (26). PP Tfh cells also play an important pathological role in the development of arthritis in the K/BxN model, and an increase in PP Tfh cells can lead to Tfh migration to systemic sites, driving auto-Ab production (29). Because of this, we examined whether P2RX7 deficiency causes an enhancement of the Tfh response in both the gut and systemic sites, leading to exacerbation of disease and auto-Ab production in the K/BxN model. As defined in previous publications, we identified Tfh cells by high PD-1 and CXCR5 expression in CD4^+^ T cells (29, 41–45). We observed strong PP Tfh induction in *P2rx7*^−/−^.K/BxN mice, analogous to what has been shown in P2RX7-deficient C57/BL6 mice [Figure 2A; (26)], however, there was no difference in the number of Tfh cells in the spleen between *P2rx7*^−/−^ and WT K/BxN mice (Figure 2A). Because one of the major consequences of P2RX7 activation is triggering cell death, and P2RX7 controls the PP Tfh population by inducing cell death (26), we examined whether differing levels of cell death in P2RX7-deficient splenic and PP Tfh cells explain the different responses to P2RX7 deletion in these tissues. Consistent with previous reports, *P2rx7*^−/−^.K/BxN PP Tfh cells had a significant reduction in cell death, as shown by decreased Annexin V staining (among intact cells that excluded Live/Dead Yellow, a membrane-impermeable dye) compared to WT K/BxN Tfh cells (Figure 2B). This method of measuring cell death has been shown to reliably distinguish early apoptotic cells from cells in late-stage cell death (46). However, the difference in percentage of apoptotic splenic Tfh cells between WT and P2RX7 deficient mice is less compared to that of PP Tfh cells (Figure 2B); i.e., the average ratio of WT to *P2rx7*^−/−^.K/BxN apoptotic Tfh cell percentage in the spleen (2.53) is less compared to the PPs (16.37). This indicates that PP Tfh cells are more sensitive to P2RX7-mediated apoptosis than splenic Tfh cells. To determine whether the increase in PP Tfh cells is a Tfh-specific effect, we also examined PD-1^lo/−^CXCR5^lo/−^ CD4^+^ T cells, a population referred to as “non-Tfh cells,” (29, 41). Our data demonstrated that there is no change in number or frequency of the non-Tfh PP population (Supplementary Figure 2A). While there is a decrease in apoptosis in the P2RX7 deficient non-Tfh population, it is again less than the decrease in apoptosis of the Tfh population (Supplementary Figure 2A vs. Figure 2B). We hypothesized that different expression levels of P2RX7 might explain the differences in Tfh cell induction and Tfh cell death we detected in the spleen compared to the PPs. Indeed, while P2RX7 expression on Tfh cells from both organs exceeded P2RX7 expression on non-Tfh cells, P2RX7 expression was much higher (>2-fold) on PP Tfh cells than splenic Tfh cells (Figure 2C). Differential expression of Bcl-6 could also potentially help explain the increased Tfh population in *P2rx7*^−/−^.K/BxN compared to WT K/BxN mice. Expression of the transcription factor Bcl-6 in CD4^+^ T cells is both necessary and sufficient to drive CD4^+^ T cell differentiation into Tfh cells, and thus Bcl-6 is considered a master regulator of Tfh differentiation (47–49). However, when we examined Bcl-6 expression in splenic and PP non-Tfh cells, we found no difference in expression levels between *P2rx7*^−/−^ and WT K/BxN mice (Figure 2D). Taken together, these data suggested that P2RX7 controls the Tfh cell population by inducing cell death, however lower P2RX7 expression levels on splenic Tfh cells limit the ability of P2RX7 to control the Tfh cell population in this systemic site.

### T Cell-Intrinsic P2RX7 Deficiency Enhances Arthritis Development and Increases the PP Tfh Cell Response

**Figure 3:**
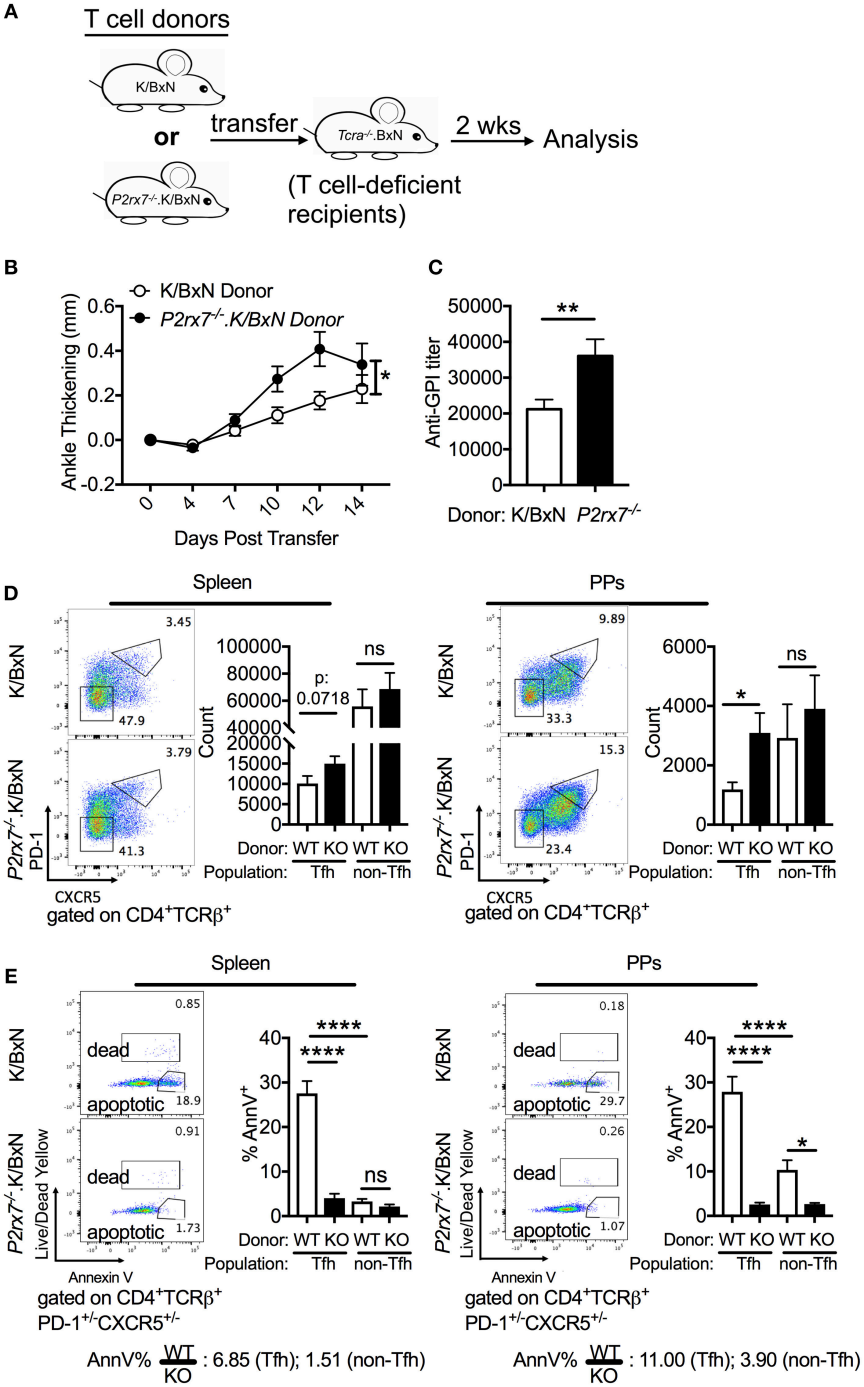
*P2rx7*^−/−^ T cells enhance autoimmunity in the SFB(–) K/BxN T cell transfer model. **(A–E)** CD4^+^ T cells were isolated from either WT K/BxN or *P2rx7*^−/−^.K/BxN donors and transferred to TCRα^−/−^.BxN recipients. **(A)** Schematic for T cell transfer. **(B)** Arthritis development was followed by ankle measurements starting at the day of transfer (day 0). Values are shown as averages of the change in ankle thickness for each individual compared to its first measurement. *N* = 20–24/group, 5 assays combined. **(C)** Auto-Ab titer as determined by ELISA against GPI. *N* = 20–24/group, 5 assays combined. **(D)** Representative plots and compiled graphs of Tfh and non-Tfh cell numbers in spleen and PPs. *N* = 15–18/group, 4 assays combined. **(E)** Representative plots of Tfh and non-Tfh cells stained for Annexin V and Live/Dead Yellow and compiled graphs of Live/Dead Yellow^−^ Annexin V^+^ Tfh or non-Tfh cells as a percentage of total Tfh or non-Tfh cells in spleen and PPs. WT = K/BxN, KO = *P2rx7*^−/−^.K/BxN. Ratios determined by dividing the average of the WT group by the average of KO group. *N* = 8–11/group, 3 assays combined. ^*^p < 0.05 ^**^p < 0.01 ^****^p < 0.0001.

To dissect whether the enhanced Tfh cell response and arthritis severity observed in P2RX7 deficiency of the whole K/BxN mouse is mediated by a T cell-intrinsic effect of P2RX7, we used the K/BxN T cell transfer system (Figure 3A). In this system, arthritis is generated by transferring K/BxN T cells into TCRα^−/−^.BxN (T cell deficient B6xNOD) mice, which allows the transferred K/BxN T cells to recognize GPI peptide presented on MHC II I-A^g7^ and develop arthritis (30, 50). *P2rx7*^−/−^ or WT K/BxN CD4^+^ T cells were transferred into TCRα^−/−^.BxN mice as described previously (29). We found that mice that received *P2rx7*^−/−^.K/BxN T cells developed worse arthritis and had increased anti-GPI auto-Abs, indicating amplified autoimmunity compared to mice that received WT K/BxN T cells (Figures 3B,C). Additionally, we found that mice receiving *P2rx7*^−/−^.K/BxN T cells demonstrated a trend toward an increase in splenic Tfh cells, and a significant increase in PP Tfh cells compared to mice receiving WT K/BxN T cells (Figure 3D). We found no change in non-Tfh cells in PPs between mice that received WT K/BxN or *P2rx7*^−/−^.K/BxN T cells (Figure 3D, Supplementary Figure 2B). Thus, as in total P2RX7 deletion, our data suggested that T cell-intrinsic P2RX7 deficiency significantly enhanced the Tfh response in the PPs. However, unlike the total P2RX7 deletion, there was a trend toward an increase in splenic Tfh cells in the spleen. Consistent with our data from the whole-mouse P2RX7 knockout system, we also found that Tfh cell apoptosis was significantly inhibited in both the spleen and PPs in mice that received *P2rx7*^−/−^ T cells compared to those that received WT T cells (Figure 3E). Also, similar to the whole-mouse knockout, the difference in average percentage of apoptotic splenic Tfh cells between WT and P2RX7-deficient mice (ratio of WT to KO = 6.85) is less compared to that of PP Tfh cells (ratio of WT to KO = 11.00). Interestingly though, the gap between the ratios of spleen and PP in the T cell transfer model (6.85 and 11.0, Figure 3E) was less when compared to the whole mouse model (2.53 and 16.37, Figure 2B). Additionally, we found that WT non-Tfh cells do not undergo cell death to the same extent as do WT Tfh cells (Figure 3E). Furthermore, the difference in apoptosis between WT and *P2rx7*^−/−^ non-Tfh cells was much less (the average ratio of WT to *P2rx7*^−/−^ apoptotic non-Tfh cell frequency was 1.51 in the spleen and 3.90 for PPs) compared to Tfh cells in both spleen and PPs (Figure 3E). These results suggested that Tfh cells are more sensitive to P2RX7-mediated apoptosis than non-Tfh cells, with the largest apoptotic difference between WT and *P2rx7*^−/−^ group occurring in PP Tfh cells.

**Figure 4:**
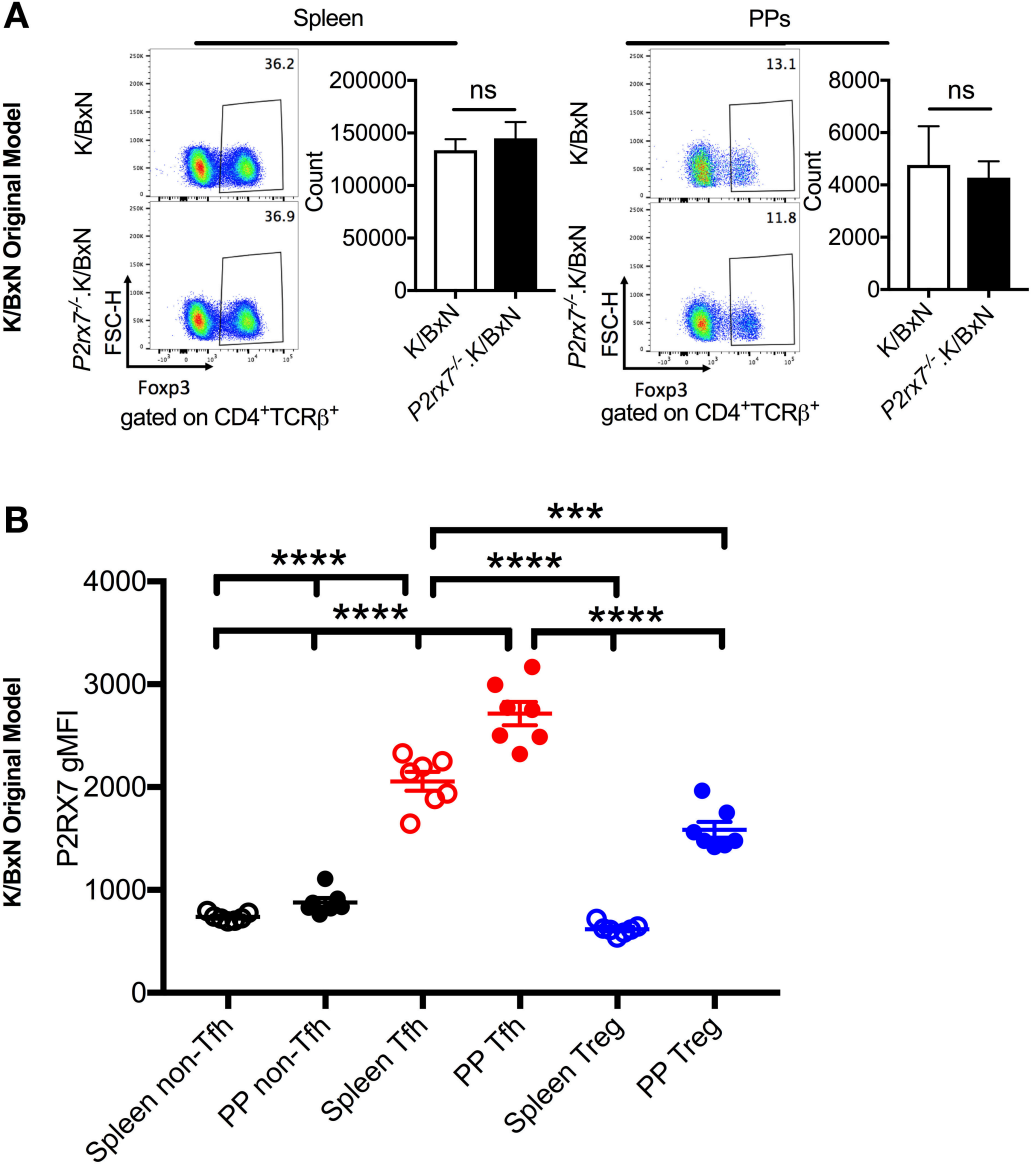
Treg numbers do not differ between SFB(–) K/BxN and *P2rx7*^−/−^.K/BxN mice. **(A)** Representative plots and compiled graphs of Treg numbers in spleen and PPs of K/BxN and *P2rx7*^−/−^.K/BxN mice. *N* = 5–11/group, 3 assays combined. **(B)** P2RX7 surface expression was detected by flow cytometry on non-Tfh, Tfh, and Treg populations from spleen and PPs of K/BxN mice. *N* = 7/group. Error bars represent SEM. ^***^p < 0.001 ^****^p < 0.0001.

P2RX7 is also associated with increased cell death in T regulatory cells (Tregs) in WT B6 mice (51, 52). To verify that changes in Treg numbers were not responsible for the increased autoimmunity we observed in the *P2rx7*^−/−^.K/BxN mice, we examined the Treg population in the K/BxN model. We found that there was no significant change in Treg cell numbers in *P2rx7*^−/−^.K/BxN compared to WT K/BxN mice (Figure 4A). We also examined P2RX7 protein levels in splenic and PP Tregs by flow cytometry with non-Tfh and Tfh cells as control groups. We confirmed that Tfh cells had much higher P2RX7 levels than non-Tfh cells, as in Figure 2C. Splenic Tregs expressed low P2RX7 at levels comparable to the non-Tfh cells (Figure 4B). Importantly, while PP Tregs expressed P2RX7 at higher levels, it was still lower than P2RX7 expression on splenic Tfh cells, which as a population, display no difference in cell numbers between WT and *P2rx7*^−/−^.K/BxN mice. This may explain why, analogous to the splenic Tfh cells, there was also no difference in PP Treg numbers between WT and *P2rx7*^−/−^.K/BxN mice (Figure 4B). This is supported by the results of another group, who found that P2RX7 mRNA, while expressed in Tregs, has the highest expression in Tfh cells (26). We additionally found that there was no significant change in Treg numbers in the K/BxN transfer model in mice that received *P2rx7*^−/−^ T cells compared to those that received WT T cells (Supplementary Figure 3). Based on these results, we concluded that Treg depletion is not a driving force in the increased autoimmunity in P2RX7 deficiency.

### SFB Do Not Further Exacerbate Autoimmune Arthritis in *P2rx7^−/−^* Mice

**Figure 5:**
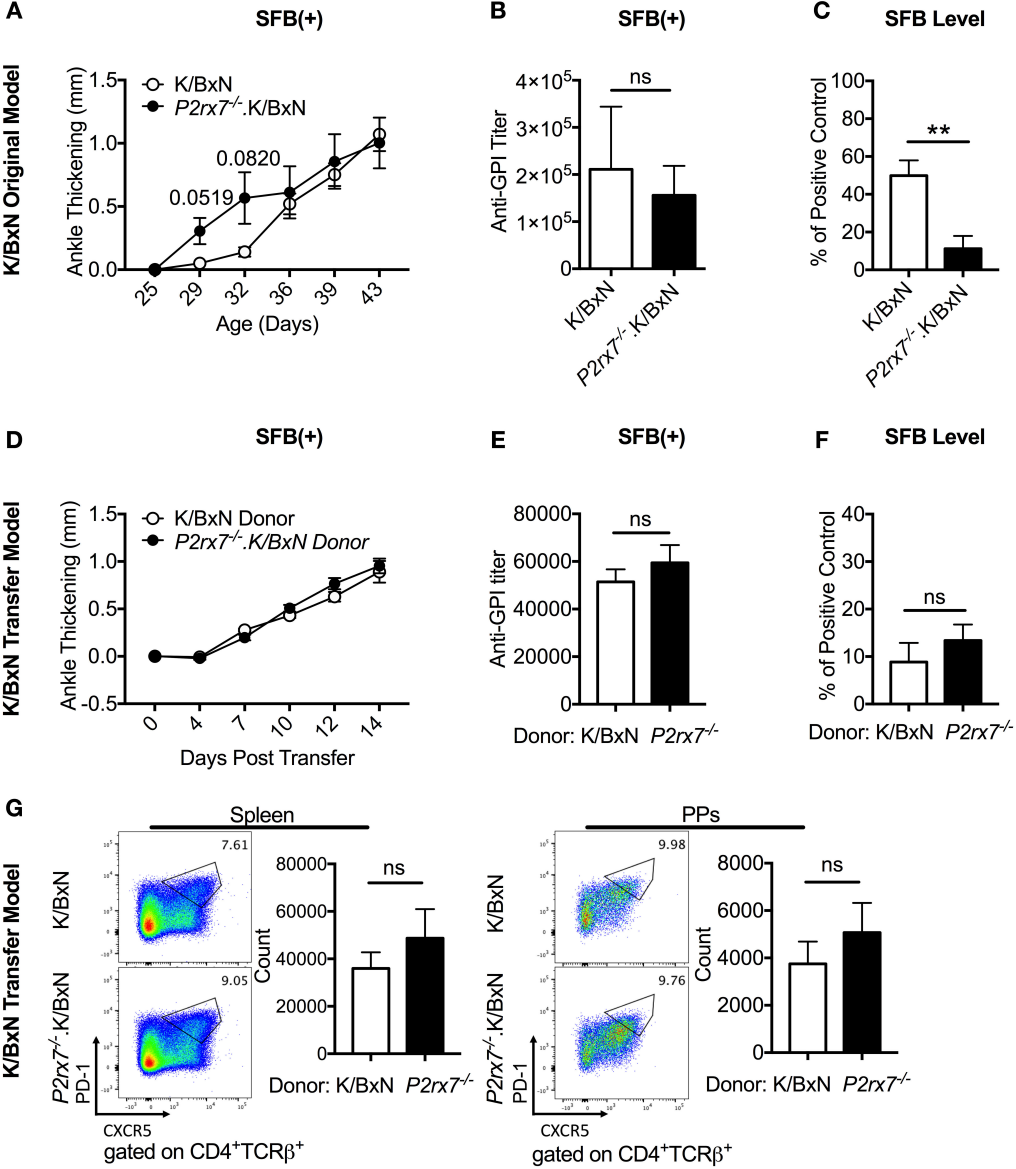
SFB colonization negates the difference in disease severity between WT and *P2rx7*^−/−^ mice in the original K/BxN model and K/BxN T cell transfer model. **(A–G)** K/BxN mice **(A–C)** or TCRα^−/−^. BxN mice **(D–G)** were gavaged with SFB 1–2 days after weaning. **(A)** Ankle thickness was followed over time for K/BxN and *P2rx7*^−/−^.K/BxN mice between ~3.5 and 6 weeks of age. Data are shown as change in ankle thickness compared to first measurement. Error bars represent SEM. *N* = 7–17/group, multiple litters combined. **(B)** Anti-GPI auto-Ab titers in serum obtained from mice 5–7 weeks old were measured by ELISA. *N* = 8–11/group, 5 assays combined. **(C)** SFB level 10 days after gavage was determined by quantitative PCR on 16S rDNA, normalized to a standardized positive control. *N* = 5–12/group, 5 assays combined. **(D–G)** SFB(+) TCRα^−/−^.BxN recipients were given either K/BxN or *P2rx7*^−/−^.K/BxN CD4^+^ cells retro-orbitally at 6 weeks of age. **(D)** Ankle thickness was followed beginning at day of transfer for TCRα^−/−^.BxN mice transferred with either K/BxN or *P2rx7*^−/^^−^.K/BxN CD4^+^ cells. Data are shown as change in ankle thickness compared to first measurement. Error bars represent SEM. *N* = 12/group, 3 assays combined. **(E)** Anti-GPI auto-Ab titers in serum obtained from the end point of each experiment were measured by ELISA. *N* = 12/group, 3 assays combined. **(F)** SFB level determined by quantitative PCR on 16S rDNA, normalized to a standardized positive control. Feces were collected at end point of each experiment (14 days after T cell transfer which was equivalent to 5 weeks after SFB gavage). *N* = 23–24/group, 5 assays combined. **(G)** Representative plots and compiled graphs of spleen and PP Tfh numbers. *N* = 11/group, 3 assays combined. ^**^p < 0.01.

Because Tfh cells have been shown to affect the gut microbiota and vice versa (26, 29), we examined whether a disease enhancing gut microbiota might combine with loss of P2RX7 to induce even greater arthritis development. To address this question, we used the gut commensal SFB, which we have previously demonstrated to exacerbate arthritis in the K/BxN system (29, 36). When we colonized K/BxN and *P2rx7*^−/−^.K/BxN mice with SFB [SFB(+) hereafter], we found that while *P2rx7*^−/−^ mice trended toward faster arthritis development than WT mice, the WT mice rapidly achieved the same level of arthritis (Figure 5A). When we analyzed anti-GPI auto-Ab titers at the experiment endpoint (~age 6 weeks old), we found no difference between SFB(+) WT and *P2rx7*^−/−^ mice (Figure 5B). We hypothesized that one explanation for the lack of difference between WT and *P2rx7*^−/−^ mice in the SFB(+) condition could be that, as demonstrated by another group, P2RX7 actively regulates IgA levels by controlling the Tfh population, permitting symbiosis with commensal gut microbes, and in *P2rx7*^−/−^ mice, SFB levels decrease due to unregulated IgA production (26). Indeed, when we performed our standard SFB level analysis 10 days after SFB gavage (~age 32 days old) by quantitative PCR in *P2rx7*^−/−^.K/BxN compared to WT K/BxN mice, we found a decrease in SFB (Figure 5C). However, the decreased SFB colonization levels in *P2rx7*^−/−^.K/BxN mice were still high enough to induce enhanced autoimmunity in our model based on previous results (29).

To further address the interactions of SFB and P2RX7, we used T cell-specific P2RX7 deletion and asked how the gut microbiota might affect the PP Tfh response. *P2rx7*^−/−^ or WT K/BxN T cells were transferred into TCRα^−/−^.BxN mice pre-colonized with SFB (see Figure 3A). Unlike in mice that were not colonized with SFB [SFB(–) hereafter], SFB(+) TCRα^−/−^.BxN mice that received *P2rx7*^−/−^ T cells displayed no change in arthritis development or auto-Ab titer (Figures 5D,E compare Figures 3B,C). These data demonstrated, similarly to what we had observed in the complete knockout model, that P2RX7 deficiency had no additive effect on disease when combined with SFB colonization. Additionally, we found that unlike in P2RX7 deficiency in the whole organism, in our T cell transfer model, T cell-specific P2RX7 deficiency did not change the SFB level after transfer (Figure 5F). As this study was done in the transfer model, to measure the transferred T cells effects on SFB colonization, we analyzed SFB level at a much later stage (two weeks after transfer which was equivalent to 5 weeks after SFB gavage) compared to the whole mouse deletion (measured 10 days after SFB gavage, Figure 5C), and thus their SFB levels had declined from initial levels, as has been noted in our previous reports (44). Nevertheless, unlike in whole mouse deletion (Figure 5C), T cell-specific P2RX7 deletion does not further reduce the SFB level (Figure 5F). This suggests that P2RX7 deficiency in T cells alone is not enough to inhibit SFB colonization levels, at least in the K/BxN arthritis model. When we further examined Tfh cells in SFB(+) transfer mice, we found that although there was a slight upward trend in Tfh numbers in mice that received *P2rx7*^−/−^ T cells compared to those that received WT cells, there was no significant difference in either the spleen or the PPs (Figure 5G).

### Up-Regulation of TIGIT Corresponds With a Reduction in Apoptosis in *P2rx7^−/−^* Tfh Cells

**Figure 6:**
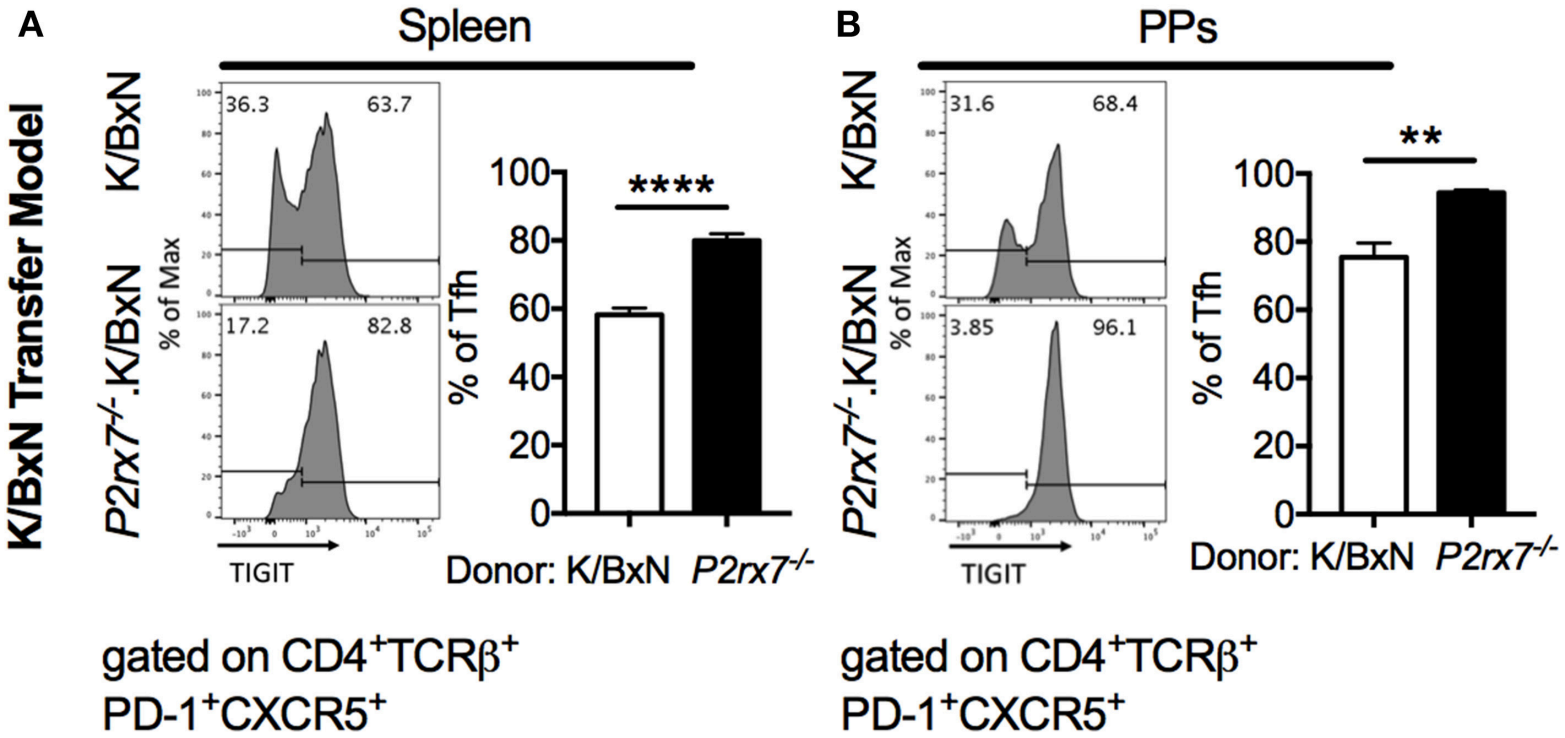
TIGIT is increased on *P2rx7*^−/−^ Tfh cells in the SFB(–) K/BxN transfer model. **(A,B)** Representative histograms and compiled graphs of TIGIT expression on **(A)** splenic or **(B)** PP Tfh cells from WT or *P2rx7*^−/−^.K/BxN CD4^+^ cells transferred into TCRα^−/−^.BxN mice. Compiled graphs show TIGIT^+^ Tfh as a percentage of total Tfh cells. *N* = 7–9/group, 2 assays combined. ^**^p < 0.01 ^****^p < 0.0001.

**Figure 7:**
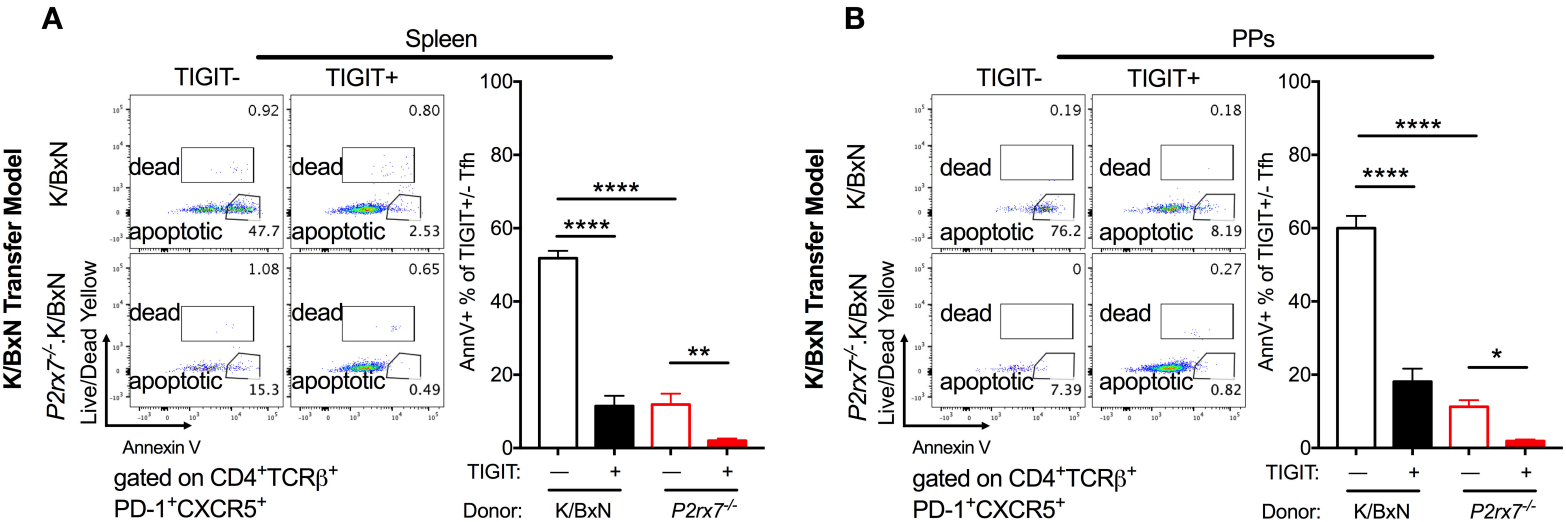
TIGIT expression on Tfh cells is associated with decreased apoptosis in the SFB(–) K/BxN transfer model. **(A,B)** WT or *P2rx7*^−/−^.K/BxN CD4^+^ cells were transferred into TCRα^−/−^. BxN recipients. Shown are representative plots and compiled graphs of Live/Dead Yellow^−^ Annexin V^+^ staining as a percentage of either TIGIT^+^ or TIGIT^−^ Tfh cells. *N* = 11–14/group, 3 assays combined. ^*^p < 0.05 ^**^p < 0.01 ^****^p < 0.0001.

Because we observed enhanced Tfh cell responses and reduced apoptotic Tfh cells in mice with both complete and T cell-intrinsic P2RX7 deficiency under SFB(–) conditions, we next investigated the molecular mechanism whereby P2RX7 exerted its pro-apoptotic effect in SFB(–) mice. In chronic infections and cancer, T cells are exposed to persistent antigens, causing T cell dysfunction, a state called “exhaustion” (53, 54). An important characteristic of exhausted T cells is that these T cells do not undergo cell death, but instead become inactive and unresponsive to stimulation (55). Indeed, activation of TIGIT, a well-known T cell exhaustion marker, up-regulates anti-apoptotic molecules and promotes T cell survival (56). Because of the reduced apoptosis in Tfh cells derived from *P2rx7*^−/−^.K/BxN mice, we asked whether P2RX7 deficiency in T cells might reduce apoptotic Tfh cells by modulating TIGIT. To investigate this question, we examined the expression levels of TIGIT on *P2rx7*^−/−^ or WT K/BxN T cells transferred into SFB(–) TCRα^−/−^.BxN mice (57). We found an increase in TIGIT expression on both splenic and PP *P2rx7*^−/−^.K/BxN Tfh cells compared to WT K/BxN T cells (Figures 6A,B). To further determine the role of TIGIT up-regulation in P2RX7-deficient Tfh cells, we examined cell death between TIGIT^+^ and TIGIT^−^ Tfh cells. We found that splenic and PP TIGIT^+^ Tfh cells, in both mice that received WT and those that received *P2rx7*^−/−^ T cells, were associated with a decrease in apoptosis, as detected by Annexin V staining among Live/Dead Yellow negative cells, compared to TIGIT^−^ Tfh cells (Figures 7A,B). In *P2rx7*^−/−^ TIGIT^−^ Tfh cells, which were already far less susceptible to cell death than WT TIGIT^−^ Tfh cells, the expression of TIGIT was associated with a further reduction in the apoptotic Tfh cell population both in the spleens and in the PPs.

## Discussion

P2RX7 has garnered a lot of interest in recent years as a potential therapeutic target for autoimmune arthritis and many other diseases (17, 19, 25). However, the results have been inconsistent and sometimes contradictory. We aimed to further elucidate the role of P2RX7 in autoimmunity by using the spontaneous autoimmune arthritis K/BxN model, along with adoptive transfers to limit the effects of P2RX7 deficiency specifically in T cells. We found that P2RX7 deficiency in the original K/BxN model led to worsened arthritis and higher auto-Ab titers. In addition, examination of Tfh cells revealed the differential impact of P2RX7 in systemic and mucosal tissues: while splenic Tfh cells changed little, PP Tfh cells increased in the absence of P2RX7, despite the fact that Tfh cells in both organs experienced a disruption of cell death. We further found that T cell-intrinsic P2RX7 ablation is sufficient to generate an autoimmune-exacerbation effect similar to that of P2RX7 deficiency in the whole mouse, i.e., an increase in arthritis, auto-Ab titers, and PP Tfh cell numbers. Many P2RX7 antagonists were designed for anti-inflammatory purposes (19). Our finding that P2RX7 deficiency mediated autoimmune arthritis counters these anti-inflammatory purposes, as we demonstrated that P2RX7 deficiency enhances rather than reduces autoimmune arthritis. We believe the conflicting beneficial or detrimental effects of P2RX7 inhibition could be based on the various etiopathogenesis in different diseases. For example, P2RX7 inhibition would likely enhance those autoimmune diseases that are mediated by auto-Abs and Tfh cells such as is seen here in the K/BxN model and in EAE models reported previously (21). In contrast, diseases that are mediated by innate immune players may be ameliorated by P2RX7 inhibition, which targets the IL-1 and IL-18 pathways (19).

Interestingly, when we colonized mice with SFB in either our K/BxN or T cell transfer model, we found that SFB colonization negated the difference observed between WT and P2RX7-deficient mice. We have previously reported that SFB enhanced the PP Tfh cell response in WT K/BxN mice and the K/BxN T cell transfer model, and PP Tfh cells are required for SFB-mediated arthritis enhancement (29). SFB-mediated disease enhancement can also be demonstrated when compared arthritis and anti-GPI titers between SFB (-) and (+) WT K/BxN mice in the current study (Figures 1A,B vs. Figures 5A,B). Thus, we suspect the lack of disease difference between SFB (+) WT and P2RX7-deficient mice is most likely because SFB do not further increase the PP Tfh cell response in P2RX7 deficient mice (compare Figure 3D and Figure 5G). We speculate that the lack of arthritis difference between WT and KO groups in the SFB (+) condition could be due to an overlapping effect between SFB and P2RX7 deficiency in enhancing arthritis development. More studies will be required to determine the exact mechanism of whether and how SFB may inhibit P2RX7 signaling to enhance Tfh cell responses and arthritis development. Our data also suggested that gut microbiota composition may account for some of the disparities in P2RX7 effects in previous studies. Notably, microbe-host interactions where the host impacts the microbiota have also been observed in regard to P2RX7 deficiency. For example, P2RX7 deficiency has been previously demonstrated to trigger an enhanced PP Tfh cell response, leading to greater IgA production by B cells to inhibit SFB colonization (26). This effect likely requires the combination of T cell-specific and B cell-specific P2RX7 deficiency, as we found that SFB colonization was reduced with whole-mouse but not T cell-intrinsic P2RX7 deficiency. Our T cell transfer data showing no difference in arthritis (Figure 5D) and SFB level (Figure 5F) between P2RX7-deficient and WT groups indicate that lower SFB levels in whole-mouse P2RX7 deficiency (Figure 5C) was not solely responsible for the lack of arthritis difference between WT and *P2rx7*^−/−^ mice in SFB(+) condition (Figures 5A,D) when compared to SFB(–) condition (Figures 1A, 3B). Future studies will be required to understand the mechanisms involved in microbiota-mediated autoimmunity in the absence of P2RX7.

**Figure 8:**
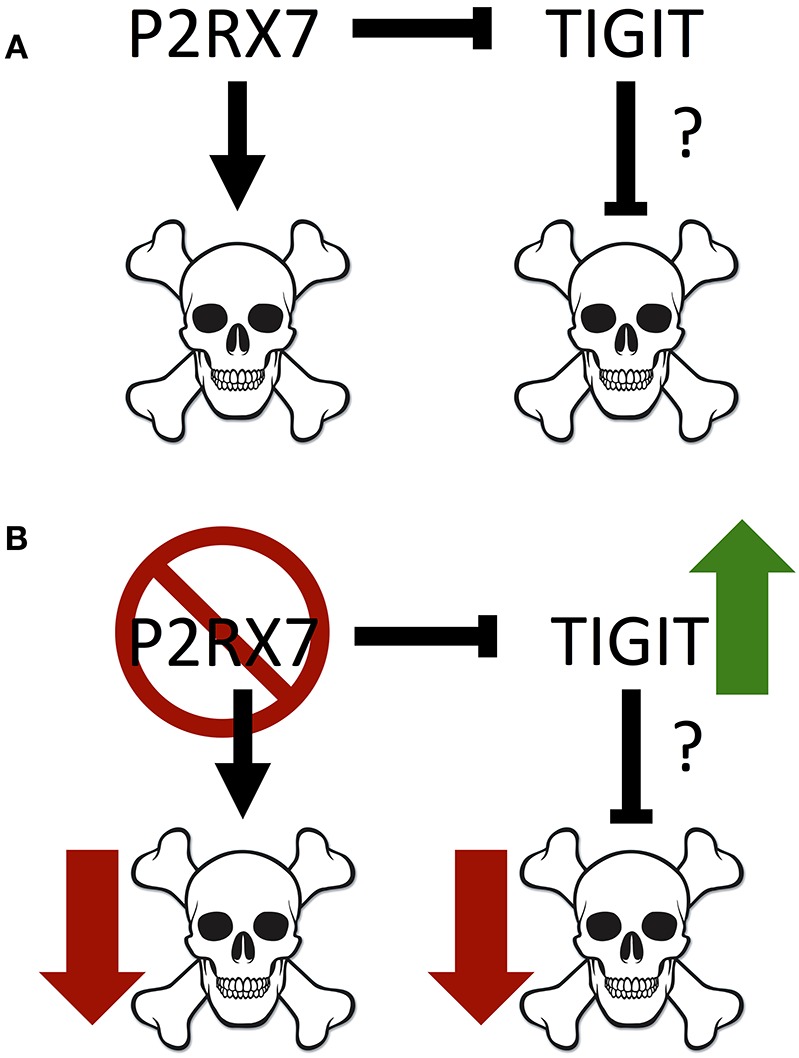
Model of interactions between P2RX7 and TIGIT, and their impact on cell death. **(A)** P2RX7 promotes apoptosis in PP Tfh cells to regulate Tfh numbers. P2RX7 may also regulate TIGIT expression on Tfh cells. TIGIT, conversely, may inhibit apoptosis to promote Tfh cell survival. **(B)** In the absence of P2RX7, apoptosis is disrupted, allowing the expansion of the PP Tfh population. In addition, the absence of P2RX7 leads to greater TIGIT expression, which may further down-regulate apoptosis.

Notably, we identified a significant increase in TIGIT expression on *P2rx7*^−/−^ Tfh cells. As mentioned earlier, activation of TIGIT up-regulates anti-apoptotic molecules and promotes T cell survival (56). In *P2rx7*^−/−^ TIGIT^−^ Tfh cells, which were already far less susceptible to cell death than WT TIGIT^−^ Tfh cells, the expression of TIGIT was associated with a further reduction of the apoptotic Tfh cell population both in the spleens and in the PPs (Figure 8). These data suggested that P2RX7 can promote cell death in the Tfh population through a TIGIT-independent mechanism (Figure 8). Our data further suggested that P2RX7 could potentially promote cell death in the Tfh population by down-regulating TIGIT and inhibiting TIGIT's anti-apoptotic effect. Clearly, more future studies need to be done to address the apoptotic regulation by P2RX7 and TIGIT. TIGIT has historically been viewed as an inhibitory molecule, which signals via ITIM domains to inhibit T cell activation (56, 57). However, more recent work has identified TIGIT^+^ Tfh cells as drivers of the immune response (58, 59). TIGIT, though it has been associated with T cell exhaustion, may have other roles as well. A pathogenic role for TIGIT has been implicated by two other groups, one of which demonstrated that a pathogenic subset of Tfh cells circulating in human RA patients' blood can be identified in part by enhanced expression of TIGIT (59), and the other of which demonstrated increased B cell costimulatory activity in a TIGIT^+^ subset of Tfh cells, which is inhibited by anti-TIGIT blocking antibodies (58).

Consistent with previous results from another group (26), we found an induction of the PP Tfh population and little change in the splenic Tfh response in *P2rx7*^−/−^.K/BxN mice compared to K/BxN mice. One explanation for the difference between the mucosal and systemic sites could be the much higher P2RX7 expression in PP compared to splenic Tfh cells (>2-fold), as detected in the whole-mouse model. This would make PP Tfh cells more sensitive to P2RX7-induced cell death than splenic Tfh cells, as the ratio of WT to *P2rx7*^−/−^.K/BxN apoptotic Tfh cells in the spleen compared to the PPs is 2.53 and 16.37, respectively, indicating greater P2RX7-mediated apoptosis in the PPs than the spleen. We have further confirmed the PP Tfh induction phenotype using *P2rx7*^−/−^.K/BxN T cells in the transfer model. Interestingly, T cell-intrinsic P2RX7 deficiency induced the PP Tfh response and it almost significantly induced the Tfh cell response in the spleen as well. This corresponded with the increased ratio of WT to *P2rx7*^−/−^.K/BxN apoptotic Tfh cells in spleen of T cell-specific deletion experiments when compared to that of whole mouse deletion experiments (2.53). These results suggested that P2RX7 deficiency in T cells alone allow T cells to be more sensitive to P2RX7-mediated deletion in systemic sites. Finally, regardless of the system, there were higher ratios of WT to *P2rx7*^−/−^.K/BxN apoptotic Tfh cells in PPs compared to the spleen. This may be due to the unique environment of the PPs, because commensal bacteria can serve as a major source of intestinal luminal ATP to activate P2RX7 (60, 61).

Similarly to Tfh cells, P2RX7 has generally been associated with increased cell death in Tregs (51, 52). Thus, the effect of P2RX7 deficiency on Tregs would likely increase Treg cell numbers, and would be unlikely to explain the increase in autoimmunity observed in *P2rx7*^−/−^.K/BxN mice. In the K/BxN autoimmune model, we did not observe a difference in Treg cell number between WT and *P2rx7*^−/−^ groups. Thus, it appears that in WT B6 mice, Tregs are more sensitive to P2RX7-induced apoptosis than the Tregs under autoimmune conditions. P2RX7 inhibits mTOR, which is a critical regulator of Tfh cells (62, 63). Activation of mTORC1 and mTORC2 expands Tfh and depletes regulatory T cells (64). Due to limitations on the scope of this study, we did not investigate the role of mTOR in P2RX7-medaited apoptosis. Undoubtedly, more studies will be required to address this important question.

In conclusion, contrary to the expected anti-inflammatory effect of P2RX7 inhibition through down-regulating innate immune responses (19), T cell-specific P2RX7 inhibition effectively enhanced K/BxN inflammatory arthritis. This effect was associated with an enhancement of the mucosal (but not systemic) Tfh cell response. These data suggest that non-selective P2RX7 antagonists may not be very effective in ameliorating autoimmune diseases, which may explain the failures in previous P2RX7-antagonist clinical trials with RA. Interestingly, a recent report suggests that P2RX7 is required for the establishment, maintenance and functionality of long-lived memory CD8^+^ T cell populations but not short-lived effector CD8^+^ T cells (65). These findings suggest that activation of P2RX7 by extracellular ATP can generate a variety of, or even opposite, outcomes based on the cell type (66). Our results illustrate a valuable lesson that cell-specific targeting of P2RX7 should be considered in order to achieve efficacy for P2RX7-related therapy.

## Author Contributions

KF, FT, and H-JW conceived and designed the study and wrote the manuscript. KF, FT, and NB designed and performed the experiments and analyzed the data. HM, IJ, KS, and NT performed SFB quantification, genotyping and ELISA analysis.

### Conflict of Interest Statement

The authors declare that the research was conducted in the absence of any commercial or financial relationships that could be construed as a potential conflict of interest.
