# Supplementary figures and images for: P2RX7 Deletion in T Cells Promotes Autoimmune Arthritis by Unleashing the Tfh Cell Response
Source: Front Immunol. 2019 Mar 19;10:411. doi: 10.3389/fimmu.2019.00411 (PMC6436202; doi:10.3389/fimmu.2019.00411)

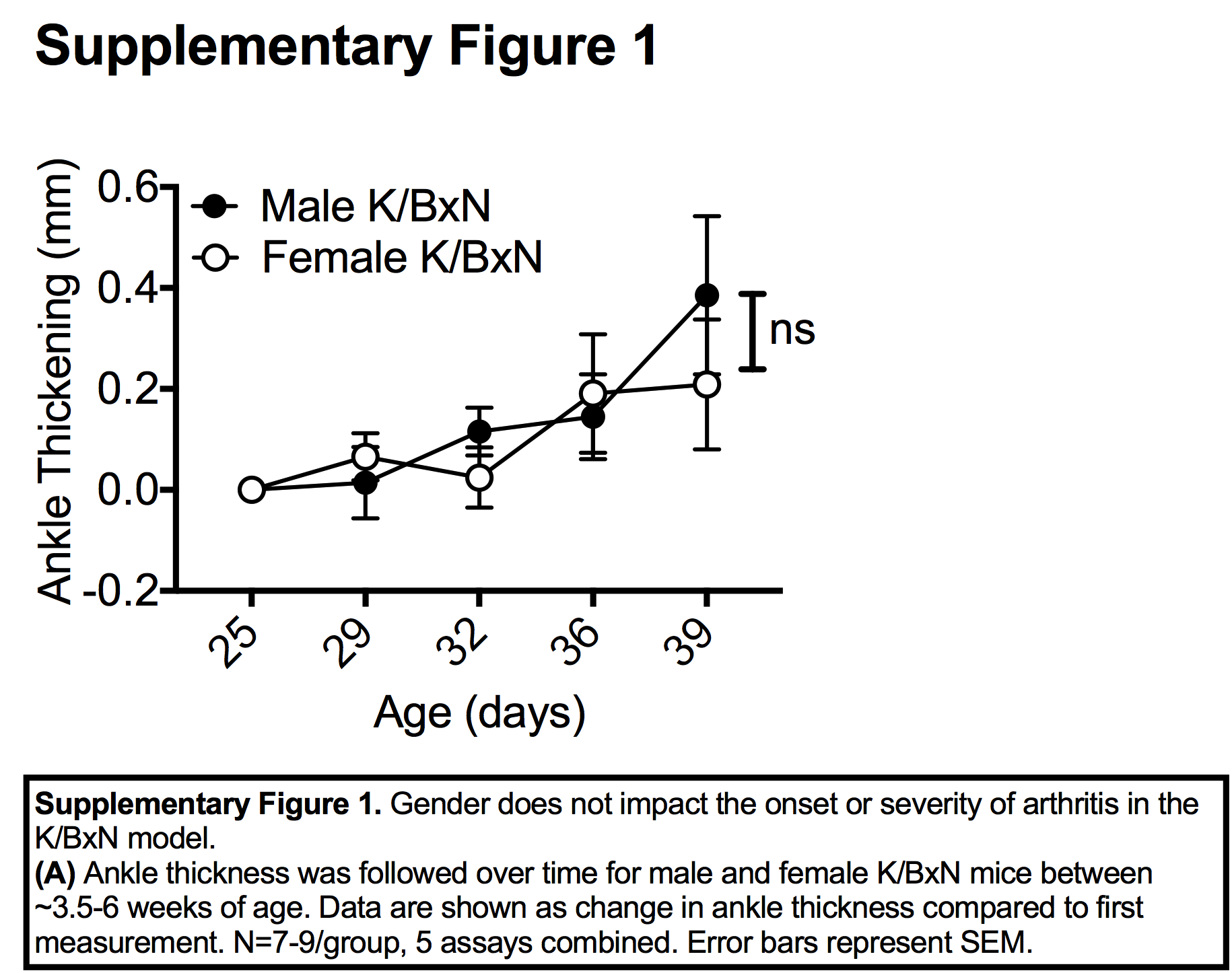

Supplement: Supplementary file 1 [file Image_1.TIFF]

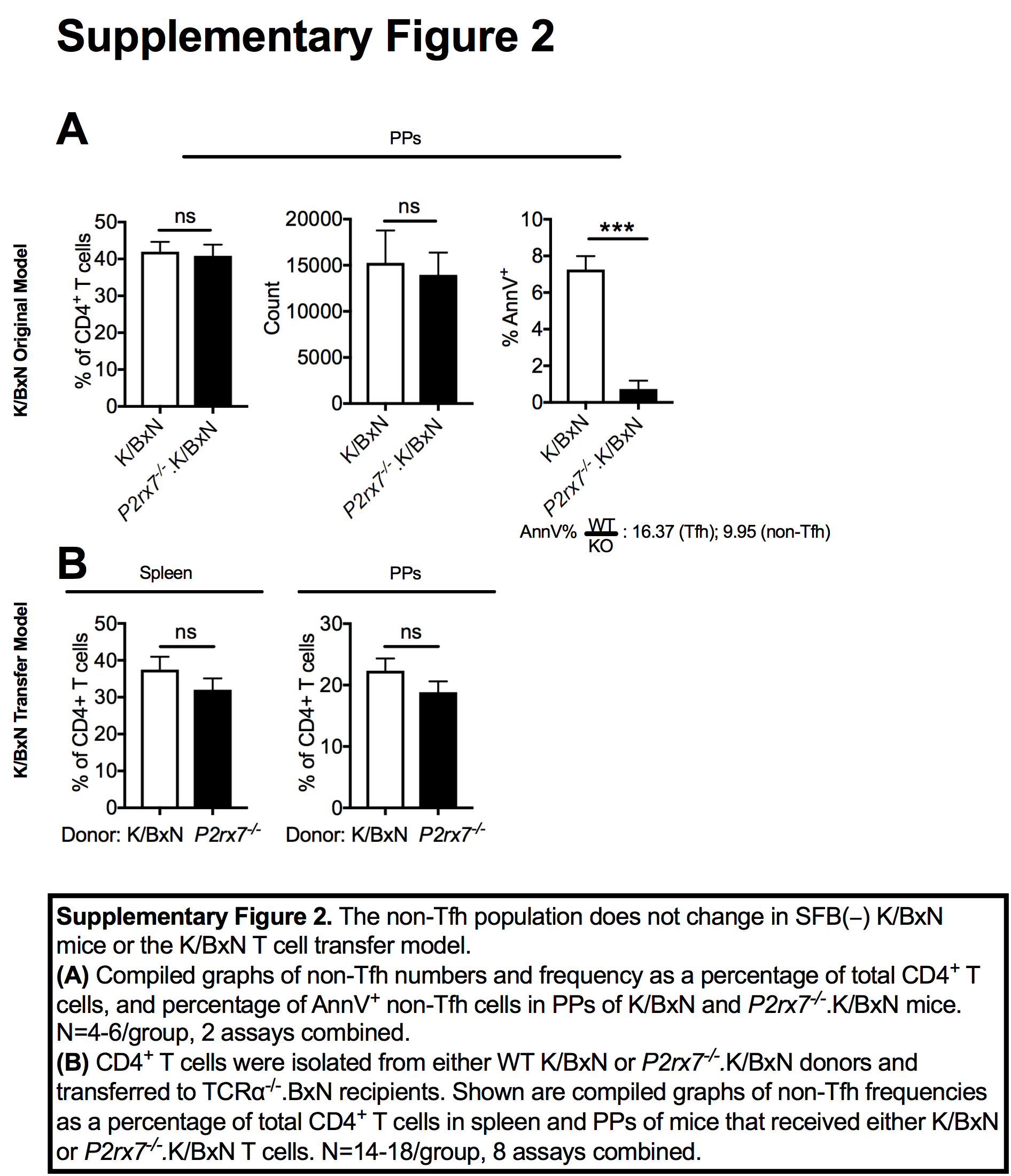

Supplement: Supplementary file 2 [file Image_2.TIFF]

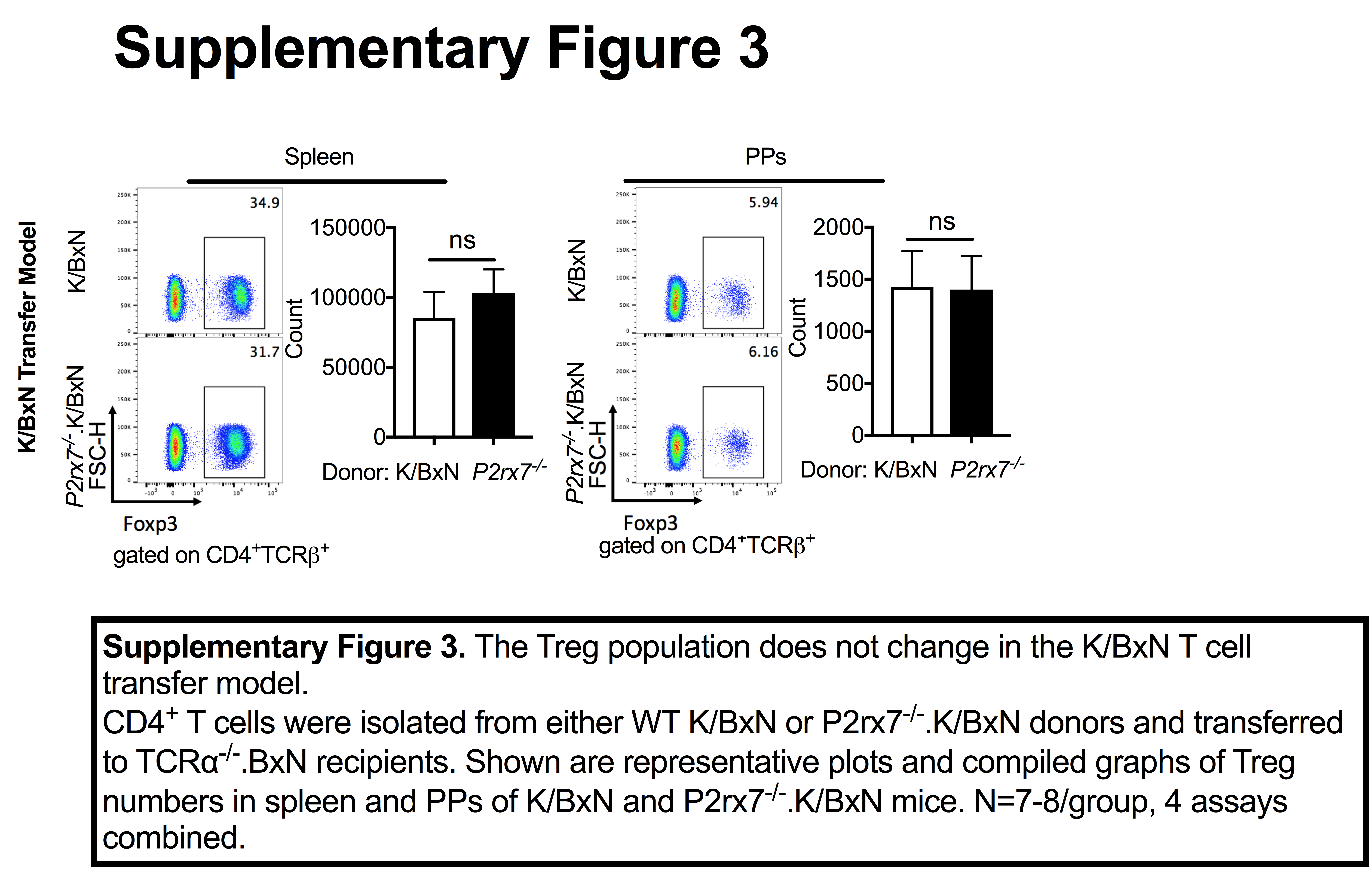

Supplement: Supplementary file 3 [file Image_3.tiff]
